# Evolutionary origin of regulatory regions of retrogenes in *Drosophila*

**DOI:** 10.1186/1471-2164-9-241

**Published:** 2008-05-22

**Authors:** Yongsheng Bai, Claudio Casola, Esther Betrán

**Affiliations:** 1 Department of Biology, University of Texas at Arlington, Arlington, TX, USA; 2 Department of Biology, Indiana University, Bloomington, IN, USA

## Abstract

**Background:**

Retrogenes are processed copies of other genes. This duplication mechanism produces a copy of the parental gene that should not contain introns, and usually does not contain cis-regulatory regions. Here, we computationally address the evolutionary origin of promoter and other cis-regulatory regions in retrogenes using a total of 94 *Drosophila *retroposition events we recently identified. Previous tissue expression data has revealed that a large fraction of these retrogenes are specifically and/or highly expressed in adult testes of *Drosophila*.

**Results:**

In this work, we infer that retrogenes do not generally carry regulatory regions from aberrant upstream or normal transcripts of their parental genes, and that expression patterns of neighboring genes are not consistently shared by retrogenes. Additionally, transposable elements do not appear to substantially provide regulatory regions to retrogenes. Interestingly, we find that there is an excess of retrogenes in male testis neighborhoods that is not explained by insertional biases of the retroelement machinery used for retroposition.

**Conclusion:**

We conclude that retrogenes' regulatory regions mostly do not represent a random set of existing regulatory regions. On the contrary, our conclusion is that selection is likely to have played an important role in the persistence of autosomal testis biased retrogenes. Selection in favor of retrogenes inserted in male testis neighborhoods and at the sequence level to produce testis expression is postulated to have occurred.

## Background

A retrogene is a processed copy of another gene. It derives from a gene through reverse-transcription of its messenger RNA and more or less random insertion into the organism's genome [1]. This duplication mechanism produces a copy of the parental gene that should not contain introns and usually does not contain cis-regulatory regions. Since retroposed gene copies most often lack regulatory regions and will not be initially expressed, they are believed to degenerate in most cases [2-4]. However, many of them are known to produce functional proteins and express in very specific pattern [5-8].

How a retrogene acquires its expression pattern and regulatory regions from the target site of insertion is a major problem in retrogene origination. In principle, retrogenes could at most carry downstream promoter elements [9,10]. Therefore, the pattern of expression of functional retrogenes should be the byproduct of the region targeted by the new insertion or of subsequent random mutations and thus we should observe diverse patterns of expression. However, this does not seem to be the case for *Drosophila *and mammalian retrogenes. Instead, they often exhibit male testis expression [5-8]. In mammals, newly generated retrogenes are often expressed in nervous system [11].

Generally, transcriptional regulation of protein coding genes in eukaryotes is achieved by the presence of a promoter (usually an upstream region where the basal transcription machinery including RNA polymerase II assembles), other cis-regulatory regions (i.e. enhancers and silencers) and the action of trans-acting factors (basal transcription machinery and other DNA-binding factors; [12]). The promoter (often called core promoter) assists transcription initiation during the basal transcription machinery assembly [13]. Tissue specific expression is usually achieved by the action of DNA-binding factors acting on cis-regulatory regions different from the promoter. However, a single sequence motif upstream of the transcription start site (TSS) can provide binding of the basal factors and polymerase and tissue specific expression; i.e. it can act as promoter and also drive tissue specific expression. A well-known sequence of this type is the 14 bp motif in the regulatory region of the *β2-tubulin *gene that is sufficient to drive late spermatogenesis specific expression in *Drosophila *[14].

Here, we computationally explore the possible routes by which emergence of retrogene expression and regulatory regions might have occurred in 94 recently described retroposed genes in *Drosophila *[5]. In particular, we examine if there is any particular set of events that explains how regulatory regions of retrogenes emerge and reveals the reason for the observed bias towards testes expression [5]. Some of the scenarios we consider here have been proposed for well-known retrogenes. Others are previously unexplored mechanisms. In particular, we address if the retrogene regulatory regions can be carried over from the parental genes [15], donated by transposable elements (TEs) [16,17], provided by nearby genes or mimicking the expression pattern of particular chromatin domains or neighborhoods [18,19].

The results show that retrogenes in *Drosophila *do not generally carry regulatory regions from their parental genes, or express in the same pattern as the closest neighboring genes, and that regulatory regions did not originate from transposable elements. However, our findings suggest that selection plays an important role in how retrogene cis-regulation emerges from the region of insertion. A fraction of retrogenes possibly survived pseudogenization by being inserted in "male-biased" neighborhoods. Interestingly, we find that this excess is not explained by insertional biases of the retroelement(s) machinery used for retroposition recently described [20]. Given this and the unexplained male-biased expression of many other retrogenes [6], we postulate that selection in favor of retrogenes with particular expression pattern and/or a few nucleotide changes fixed under selection may need to be invoked to produce the regulatory regions and patterns of expression observed for the studied retrogenes.

## Results

### Did regulatory regions of retrogenes originate from their parental genes?

Retrogene regulatory regions can originate from an aberrant upstream transcript of the parental gene that was longer at its 5' end, and contained the regulatory region(s) of the parental gene. The mammalian retrogene *Pgk-2 *is believed to have attained its initial expression by this mechanism and additional changes in the regulatory region(s) determined its male germline specific expression. This conclusion is supported by the position of the direct repeats that flank retrogene insertion and by the fact that there is possibly as much as 860 bp of 5' flanking sequences that show conservation between *Pgk-1 *parental gene and *Pgk-2 *retrogene known to contain the parental regulatory region [15].

Below, we examine whether the *Drosophila *retrogenes regulatory regions originated in this way or originated from a normal transcript of the parental gene that contained internal (i.e. downstream) regulatory regions.

To address the first possibility, we looked for similarity in the upstream region of both retrogene and parental gene. We used the blastn program [21] to search for nucleotide similarity between the 500 bp of the 5' flanking regions of the 94 retroposed gene copies described by Bai et al. [5] and the corresponding 500 bp of the 5' flanking regions of their parental genes. No hit or clear similarity was found in any comparison with the exception of a small segment of 14 bp hit between the pair *CG12334 *and *CG32672*. While these analyses are unlikely to reveal homology for ancient retroposition events, we did not find evidence of the upstream regions being carried over for any of the young (less than 15 My old) retroposition events [5]. Young retrogenes do not show sequence similarity extending upstream of the 5'UTR of their parental genes in *D. melanogaster *(data not shown). However, the upstream regions of 6 out of 6 of the parental genes give significant multiple blastn hits between *D. melanogaster *and *D. yakuba*, which indicates that conserved regulatory regions are detectable by homology search over this time scale (data not shown).

We addressed the next alternative by exploring tissue co-expression of parental and derived genes. Our previous work [5] highlighted that parental genes tend to be expressed in more tissues than retrogenes in terms of the average number of cDNA/EST library hits, with a higher percentage of parental genes expressed in all tissues besides adult testis. Indeed, 53% of retrogenes versus 42% of parental genes are expressed in adult testis. So, in order to address to what extent these retrogenes expressed in testis inherited internal regulatory regions from their parental gene transcripts, we first analyzed retrogene *vs. *parental gene co-expression in adult testis. We found that 24 retrogenes are expressed in testis derived from parental genes with at least one EST/cDNA present in the testis library, a number very close to the expected 20 retrogenes calculated as the product of what fraction of retrogenes are expressed in testis and what fraction of parental genes are expressed in testis (shown above). In addition, a detailed comparison of the upstream motifs between each retro-parental gene pair with co-expression in adult testis did not reveal any case of motif carry-over identified as a shared-overrepresented motif in the species that contain these genes (data not shown).

### Are retrogenes and neighboring genes co-expressed?

The local genomic environment has been shown to have effect on the spatial/temporal activity of genes, mainly because of the chromatin organization of flanking regions that might influence regulatory regions of genes [18,19]. In the *D. melanogaster *genome, it has been proposed that transcriptional co-regulation of clusters of co-expressed genes in male testis could be due to existence of chromatin domains [18,19]. Detailed study of a cluster of five male testis genes showed that they lay in open chromatin in male testis compared to adjacent regions and somatic cells [18]. With these observation in mind, we explored if retrogene pattern of expression overlaps with its flanking genes and if retrogenes that express in male testis are within previously described male testis clusters or neighborhoods [22]. We reasoned that if a retrogene is inserted in a neighborhood where many genes express in a particular tissue, that could provide context to the retrogene, i.e. the expression pattern for this retrogene might mirror the neighboring genes expression. It is also arguable that since retrogenes are generated in the germline they might insert in germline open chromatin and because of that mimic the expression pattern of the neighboring germline genes.

**Figure 1:**
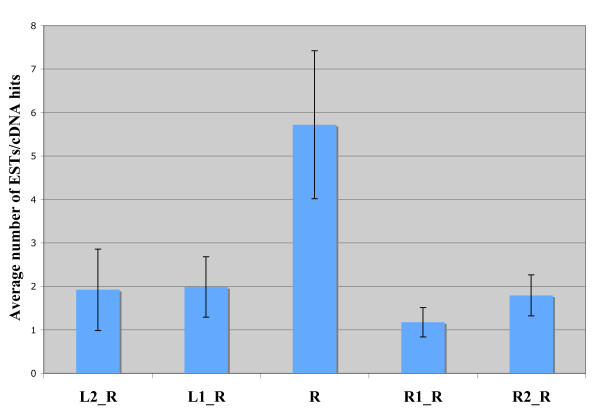
**Adult testes expression level for the retrogene (R), and two flanking genes on each side (left (L) and right (R)).** Standard error bars are given.

First, we compared the expression pattern of retrogenes and their neighboring genes, in particular the closest two genes on each side, determining the presence of gene-specific mRNAs and/or ESTs in available *D. melanogaster *libraries (see Methods). We consider that a retrogene shows co-expression with the neighboring genes if at least one of the four genes flanking the retrogene (two on each side) is expressed in one of the same tissues. We observed 62.5% of co-expression for retrogenes in adult testes (30/48). We compare this co-expression with the co-expression observed after sampling at random 48 testis expressed genes 1000 times and reveal that only 8 samples have this level of co-expression or smaller (i.e. P = 0.008). Thus, we conclude that the testis co-expression between retrogenes and their neighboring genes is significantly smaller than the one observed in "typical testis expressed genes" and that this level of co-expression is unlikely to be consistently related to the retrogenes close genomic environment. In addition, the level of expression of retrogenes in adult testes is not explained by the neighboring genes, i.e. in average all four neighboring genes are expressed at significantly lower level than the retrogenes (P = 0.0067 under the test of one-way ANOVA (Miller 1997)). Since the difference between the mean value of adult testes library hits for 91 retrogenes with any of their neighbors is more than the least significant difference value at 5% level, the level of adult testis expression for retrogenes is significantly different from (higher than) any of its two neighbors on either side (see Figure 1). However, "testis-expressed" retrogenes do not look either like a random set of genes in the genome with respect to their level of testis co-expression. We observe that after sampling at random 48 genes from the genome 1000 times the probability of this level of testis co-expression (30/48) or higher is 0.015.

We also studied expression bias in retrogene and their neighboring genes by using FlyAtlas microaarray data [23] and Parisi et al. microarray data [22]. For the FlyAtlas data, we looked at each retrogene and its closest two neighboring genes with respect to their unique pattern of expression and predominant expression in testis as defined by Chintapalli et al. [23]. Results are shown in Additional file 1. See also Additional file 2 for all the expression data. The genome wide expression pattern revealed that there are 1,317 genes that show unique testis expression out of the total 18,770 genes [24]; in comparison, many more retrogenes (Additional file 1) show testis unique expression (34 out of 94; Fisher's exact test P < 10^-6^). However, this bias is not explained neither by left nor right neighboring genes that show significantly less unique testis expression and that do not differ from the genome wide pattern (Fisher's exact test P_left genes_= 0.1448 and P_right genes _= 0.0759). Similar results are obtained when we compare number of genes that show predominant expression in testis as defined by Chintapalli et al. [24] (data not shown). Similarly to what we observed above for the cDNA/EST data (Figure 1), level of expression in testis for retrogenes is not explained by neighboring genes when we compare average mRNA signal in the FlyAtlas data (data not shown). Level of expression in retrogenes is on average significantly higher than for any of the flanking genes (P < 10^-5 ^in a one way ANOVA). From these data retrogenes are estimated to express between 2.4 and 3.7 times higher on average than neighboring genes.

We then studied retrogene and neighboring genes co-expression using the data set from Parisi et al. [22], 6.5% (930/14233) of the genes of the genome have "male-biased" expression, including twenty-eight retrogenes. In this case, we examined the closest two genes on each side, determining the presence of "male-biased" expression in the neighboring genes of "male-biased" retrogenes. Interestingly, we noted that the percentage of retrogenes that show "male-biased" expression using Parisi et al. data was much higher (36%; i.e. 28 out of the 77 retrogenes described by Bai et al. 2007 that were included in the array) than the genome wide (6.5%). This again supports the bias towards testis expression in retrogenes observed in previous studies using EST data [5,6].

In the co-expression analyses using microarray data, we observed that five of these "male-biased" retrogenes (17.9%) show a co-expression bias (i.e. they have at least one "male-biased" neighboring gene). After sampling at random 28 "male-biased" genes 1000 times we observe only 33 genes with this level of co-expression or smaller (P = 0.033), confirming again that testis-biased expression for retrogenes does not show the level of correlation with the neighboring genes expected for a "typical male-biased" gene. However, "male-biased" retrogenes do not look like a random set of genes in the genome either with respect to their level of co-expression bias with neighboring genes. In particular, we observe that after sampling at random 28 genes from the genome 1000 times the probability of this level of bias (5/28) or bigger is 0.007.

From this set of analyses we infer that on average the level of co-expression of retrogenes with their neighboring genes neither matches the expected for testis expressed genes nor the one expected for genes selected at random from the genome. The level of co-expression for retrogenes is somewhere in between. This could be explained by a small fraction of testis-expressed retrogenes being in testis neighborhoods as revealed below.

Looking at previously reported testis domains, we noticed that four testis expressed retrogenes previously reported [5,6] are located in the significant testis neighborhoods described in figure 8 of Parisi et al. [22]: *CG3162*, *RpL37b *(*CG9873*), *CG10839 *and *CG13340*. Very interestingly, given that the authors only reported four very significant testis neighborhoods (see fig. 8 of Parisi et al. [22]) and that they cover 1.3% of the euchromatic genome (1.57 Mb/120 Mb), having 4 retrogenes in these regions out of the 100 previously described [5,6] is significantly more than expected by chance (X^2 ^= 5.6816; P = 0.0171).

To argue that the expression pattern for these four retrogenes was provided by the context and that this was selected, the neighborhoods need to predate the new gene insertions. Synteny conservation analysis of the genes located in these expression neighborhoods across *Drosophila *species using the University of California at Santa Cruz Genome Browser Database [24] revealed that the organization of the three neighborhoods predates the radiation of the *Drosophila *genus (data not shown). Since 3 of the retrogenes are also older than the radiation of the *Drosophila *genus, we cannot infer if they originated before or after the neighborhoods. However, in the particular case of *RpL37b*, the retrogene neighborhood is older than the retrogene [5]. Therefore, it remains possible that for few of our retrogenes the neighborhoods provided a chromatin context that helped them to acquire testis expression, if we assume that the expression patterns of the genes in the neighborhoods have remained unchanged.

The observed significant proportion of retrogenes in testis neighborhoods could be either explained by biased insertion of retrogenes or by selection retaining retrogenes when they insert in particular genomic environments. It is believed that retrogenes use the machinery of transposable elements for their insertion. In *D. melanogaster*, TE insertions are affected by several factors, including recombination, genome compactness, gene expression, and presence of clusters of co-expressed genes [20]. In particular, the number of TE insertions on the X chromosome is higher than expected. Interestingly, TEs tend to insert close to genes expressed in male and female germline [19]. On the contrary, retroposed genes show a bias for autosomes [6], many are male biased [6], and above data] and are not consistently part of gene neighborhoods with the same expression bias (above data), with the possible exception of few retrogenes in testis neighborhoods.

### Did transposable elements provide regulatory region(s) to retrogenes in *Drosophila*?

Transposable elements could provide regulatory elements to retrogenes [16]. They are genome sequences able to make copies of themselves or transpose through transcription and translation of the proteins needed for copying or transposing. This transcription is often driven by existence of internal regulatory regions [16]. Some TEs and viruses show biased expression patterns being highly transcribed in tissues like male and female germline helping their increase in copy number and transmission to the next generation [25,26]. These regulatory regions can become regulatory regions of host genes [16,27]. There could also be a bias for the insertion of retrogenes in the same regions where retroelements insert given that they are known to use the same machinery [28] or for both of them to insert in open chromatin, inserting thereby in the same regions [29]. This could put transposable elements and retrogenes in close proximity possibly propitiating the donation of regulatory elements from transposable elements to retrogenes. In mouse, there is, in fact, one example of a retrogene whose regulatory region originated from the promoter of a LINE1 retrotransposable element. The *PMSE2b *gene is intronless and encodes the β-subunit of the proteasome activator PA28. Its parental gene *PMSE2 *contains introns. Authors observed that the intronless gene was inserted into a LINE1 element. The luciferase assays proved that the retroelement drives the current expression of the gene [17]. In this work, we computationally explore if there are any remnants of TEs that could have donated regulatory regions to the retrogenes and explain their male testis expression bias. We investigated the presence of TEs or remnants of TEs in the retrogenes UTRs and in their 2 kb upstream and downstream regions, and compared them with the same regions of the whole data set of "canonical" (non-retroposed) genes reported in FlyBase (Table 1).

**Table 1:** Transposable elements inserted nearby of 97 retrogenes and 13375 "canonical" genes

Gene region	Gene set^a^	Genes associated with TEs^b^	Proportion (%)	Fisher's exact test
5'UTR	Retrogene	1	1.03	P = 1.0000
	"Canonical" genes	97	0.73	
3'UTR	Retrogene	2	2.06	P = 0.2699
	"Canonical" genes	139	1.04	
2 kb upstream	Retrogene	7	7.22	P = 0.7198
	"Canonical" genes	1189	8.89	
2 kb downstream	Retrogene	12	12.37	P = 0.1338
	"Canonical" genes	1085	8.11	

^a ^"Canonical" genes refers to FlyBase genes annotated in the *D. melanogaster *release 3.2.1 [52].

^b ^TEs: transposable elements

Three main aspects of TEs-retrogenes relationship emerged. First, we found few TE inserted in the coding regions or UTRs of retrogenes, which confirms previous studies showing that transposable elements are rarely contributing to the coding sequence and the UTRs of genes in *Drosophila *[30].

Second, a very similar proportion of TEs is inserted in the UTRs and upstream-downstream regions of retrogenes and "canonical" genes (Table 1) revealing that similar evolutionary forces at are play in both types of genes. There is not support for a biased insertion of retrogenes in the same regions where retroelements insert.

Third, TE insertions have been detected within UTRs and in the proximity of about one fourth of the retrogenes (22.7%, see Table 1), and mainly in the 3'UTR and 2 kb downstream region (14.4%). Given that promoters and often other cis-regulatory regions are expected to be 5' of the gene, these transposable elements likely played a small role, if any, in the formation of retrogenes regulatory regions. Moreover, after comparing the age of *D. melanogaster *retrogenes as recently estimated [5] to the age of nearby TEs deduced by their conservation in other *Drosophila *species (see Methods), we observed that retrogenes are often much older than the nearby TEs (Additional file 3). As it is believed that new gene copies in the fruit fly are destined to be quickly lost if they do not acquire a new function, i.e. very few pseudogenes are found in this genome [31], these TEs can at most have affected the expression pattern of already expressed (functional) retrogenes. There are only two remnants of TEs older than proximal retrogenes (one upstream of the gene *Fad2 *and one downstream of the gene *ran-like*), both representing internal fragments of a ROO long terminal repeat (LTR) retroelements, 58 bp and 90 bp long respectively. They are located in larger conserved regions, which can be involved in the regulation of retrogenes or other surrounding genes activity.

However, this observation is in evident contrast with the reported age of ROO LTR elements in *D. melanogaster*, which are very young and at least in some case still active [32]. Comparing the sequence of the two ROO fragments close to *Fad2 *and *ran-like *we found that they both correspond to the region between nucleotide 640–725 of the ROO full-length consensus sequence deposited in Repbase [33], which lies between the LTR and the ORF of the retrotransposon and it is likely that these conserved sequences have been misannotated as ROO-derived fragments, in both coding and non-coding genomic regions, due to the accidental similarity between their low-complexity regions.

Overall, TEs seem to have possibly donated very few or no regulatory regions that determined the retrogene survival. It should be noted, however, that given the age of fruit fly retrogenes, 68% of which originated before the Drosophila-Sophophora subgenus divergence [5], it is not possible to assess how much ancient and unrecognizable transposable elements might have contributed to the regulatory sequences of retroposed genes.

## Discussion

In recent years, many functional retroposed copies of genes have been discovered in different organisms [5,7,34]. The presence of such functional retrogenes raises the question about how their expression is regulated, as in most cases they should not have inherited specific regulatory regions from their parental genes. Here, we studied the evolutionary origins of cis-regulatory regions of retrogenes in *Drosophila*.

A first aspect to discuss is the fact that we did not look at chimeric retrogenes. A particular type of chimeric retrogenes are those that form after the retrogene insertion occurs downstream of another gene (donor of regulatory region and additional exons) and produce a chimeric transcript. This will occur more likely if the donor of the regulatory regions is a member of gene families where some redundancy is present, or in recently duplicated genes, where the retrogene insertion would affect to a less extent crucial existing functions. In these cases, the upstream gene regulatory region is used producing a chimeric protein. There are several cases of this type of chimera in *Drosophila*. The first one described was *jingwei*. *Jingwei *[35] is a chimeric gene derived from *ymp *and a retrogene originated from the *Adh *parental gene. In *jingwei*, the regulatory regions are inherited from the *ymp *gene, and as expected the expression pattern of this chimeric gene mirrors that of *ymp *[35]. In the case of *jingwei*, male specific expression is due to its fusion with *ymp *[35]. However, in our retrogene list [5], it is likely that none of the retrogenes is chimeric with other known genes because in our annotation procedure, we required the pairs parental/retrogene to align over at least 70% of the proteins encoded by each gene. At least for the 65 retrogenes that have an annotated 5' end, we have support for them not being chimeric with other genes. Therefore, our analyses focus on the study of the evolutionary origin of cis-regulatory regions of "non-chimeric" retrogenes in *Drosophila*.

In this work, we address a particular aspect of the evolutionary origin of regulatory regions in "non-chimeric" retrogenes: the origin of testis expression. Our previous results [5] and present results suggest that *Drosophila *retrogenes show a bias for transcription in adult testis. First, we try to understand if this expression originates from the parental transcript or motifs inherited from parental genes. We observe no evidence to support that the retrogene carries any testis regulatory region from the parental gene. There is no bias for overlapping expression patterns in parental gene and retrogene and no internal motifs are shared between testis expressed parental gene and retrogene pairs.

We conclude that retrogenes regulation of expression, in particular their testis-specific expression, seems to originate mostly independently from their parental genes. However, the possibility remains that regulatory regions responsible for the expression in adult testis of parental genes could have been "transmitted" to their retrogenes, but successively lost by either the parental genes or retrogenes. Given that most retrogenes emerged more than 10 Mya [5], and the high turnover of transcription factor binding sites in *Drosophila *[36], transmission and loss of regulatory regions remains a possible mechanism for old retrogenes.

We also show that transposable elements do not appear to be a frequent source of regulatory regions for retrogenes although we cannot rule out the possibility that ancient transposable elements provided regulatory motifs to nearby retrogenes. In addition, despite the fact that retrogenes use machinery of transposable elements, they do not follow the transposable elements genome location pattern recently described in detail [20]: high density in the X chromosome and consistent bias insertion close to female and male germline expressed genes. On the contrary, retrogene set shows an excess in the autosomes [5,6], male biased expression and no clustering or co-expression with close flanking genes in testis except for few genes that are in testis neighborhoods. This reveals that selection probably has a predominant role in the case of retrogenes. Indeed, we do not observe a significant excess of retrogenes on the X chromosome but on autosomes [5,6].

In the four retrogenes detected in highly significant testis-biased neighborhoods, different forces are likely to have contributed to their localization. Testis-biased neighborhoods could represent a positively bias target for retrogenes because they are open chromatin in male germline [20] but the fact that "male biased" expression is a feature of many retrogenes in addition to these four would support the thesis that retrogenes express in testis were favored by selection. It has been suggested that there could be promiscuous expression of genes in male germline due to the high levels of RNA polymerase in spermatids in rodents [37]. However, in Drosophila, it is known that there is a very specific change of expression profile in male germline (i.e. many genes are turned off and on in a tightly regulated way; [38]). In many instances, male-specific gene family member or alternative transcription start sites for many genes are used [39,40]. This is attained through epigenetic changes and testis specific transcription machinery [41]. So, it seems that, in these four genes and the other testis retrogenes, a particular core promoter or other regulatory regions for testis expression will have to originate to be expressed in this pattern [41]. This is consistent with the well-defined transcription start site and testis-specific expression and function of many of the retrogenes [42-48]. Therefore, the chromatin context and/or high levels of RNA polymerase in spermatids might not be enough to explain testis expression and the origination of a testis cis-regulatory region through few substitutions might be needed to express retrogenes consistently in such a pattern.

We propose that the complex interplay between where the retrogene is inserted, and selection in favor of retrogenes that inserted either in particular neighborhoods or that acquired particular patterns of expression by means of few changes in the region of insertion might lead to the emergence of the regulatory regions that currently drive male expression in retrogenes. This last alternative was suggested for *Dntf-2r *[46] wherein a short region upstream of the gene has similarity to a testis specific element that was not present before *Dntf-2r *insertion. In mammals it has been possible to reveal these proposed selective pressures by comparing expression patterns of expressed retropseudogenes and functional retrogenes [8] and revealing the biases for the functional ones. In *Drosophila*, retropseudogenes are scarce [31] and we do not possess this kind of data. However, as discussed above, we still have a basis to postulate that positive selection has been acting during the origination of retrogene regulatory regions. The regulatory regions of *Dntf-2r *and several other young retrogenes are currently being experimentally studied in our laboratory in order to test the validity of this hypothesis.

## Conclusion

We study how promoter and other cis-regulatory regions of retroposed copies of genes may have originated. We in particular focus on explaining how their male testis expression arose. Most hypotheses investigated are rejected: (1) we infer that retrogenes do not generally carry regulatory regions from their parental genes, (2) close neighboring genes do not usually share regulatory regions with retrogenes and (3) transposable elements do not appear to substantially provide regulatory regions to retrogenes. Interestingly, we find that there are four retrogenes in male testis neighborhoods but not a consistent bias for co-expression with neighboring genes. We conclude that retrogenes' regulatory regions do not represent a random set of existing regulatory regions. On the contrary, selection in favor of particular insertions and at the sequence level to produce testis expression is postulated to have occurred.

## Methods

### Genes used in this study

The genes we looked at in this study of retrogene regulation are the retrogene pairs described by Bai et al. [5]. The set of 94 retroposition event previously described with its assigned "parental" gene (gene from which the retrogene originated) has been used. In three instances, retrogenes tandemly duplicated [5] and we explore these additional genes (amounting to 97 retrogenes) in particular analyses. We also used information of four neighboring genes. These are the two closest left and right neighboring genes for the retrogenes and parental genes. We did that independently of their strand orientation. If the gene (retrogene or parental gene) was overlapping with another gene, the overlapping gene was assigned as the nearest neighboring gene on both sides.

### Tissue expression analyses

The approach described in previous work [5] was used here to conduct the analyses for tissue expression. In short, we downloaded *D. melanogaster *ESTs & cDNA database (October 2003 release) locally from the Berkeley *Drosophila *Genome Project. We queried these databases using Blastn [21] with our retrogene, parental gene and neighboring gene data set to infer expression of a particular gene. This type of expression data allows for the assertion of expression of duplicate genes without the confounding effects of sequence similarity between duplicates [6]. Microarray data from Parisi et al. [22] for male germline-biased expression was also used for comparison and because it can give additional quantitative information. Tables and figures containing male germline-biased expression data were inspected. FlyAtlas data [23] was also inspected for unique expression in testis and predominant expression in testis for retrogenes and neighboring genes. Interestingly, all the different approaches gave consistent results (see Results section).

### Transposable elements distribution nearby and within retrogenes and "canonical" genes

We used the *D. melanogaster *April 2006 assembly (dm3) at UCSC Genome and Table Browser Databases [49,50] to detect transposable elements (TEs) and TE fragments longer than 50 bp in untranslated regions (UTRs) and 2 kb-long flanking regions of retrogenes and other ("canonical") FlyBase-annotated genes. Separate custom tracks were created for the retrogenes and the canonical genes 5'UTR, 3'UTR and 2 kb regions upstream and downstream regions. The genomic coordinates of TEs contained in these regions have been obtained intersecting each above track with a TEs-only custom track developed filtering the table field RepClass (RepeatMasker track of the Variations and Repeats group) with the words "LTR", "LINE" and "DNA".

The approximate age of the detected 22 TEs was established determining the presence of orthologous elements in six other *Drosophila *species (*D. simulans*, *D. yakuba*, *D. ananassae*, *D. pseudoobscura*, *D. virilis*, and *D. mojavensis*,) using the Comparative Genomics track on the UCSC Genome Browser. A similar approach was followed to assess the age of each retrogene and parental gene [5], and allowed a comparison of relative age for TEs and associated retrogenes. The conserved ROO copies were selected by intersecting a specific ROO custom track (we filtered the RepeatMasker track of the Variations and Repeats group with the word "ROO_I") with the "Most Conserved" track, allowing 80% overlap.

### Shared motif analyses in parental genes and retrogenes

We looked for motifs in a defined putative promoter region between -100 and +40 relative to the TSS for genes with annotated 5' UTRs. To find shared sequence motifs in this putative promoter regions of retrogenes and parental genes, we *blastned *[21] each retroposition pair and visually inspected each possible carryover case.

## Abbreviations

Mya: millions years ago; TSS: Transcription start site; Transposable element: TE; Untranslated region: UTR; bp: base pairs.

## Authors' contributions

EB conceived the questions. YB and CC acquired the data. YB, CC and EB analyzed and interpreted the data. EB wrote the article. YB and CC participated in drafting some sections of the manuscript and in revising its content.

## Supplementary Material

Additional file 1 Summary of unique and predominant expression of retrogenes and neighboring genes from FlyAtlas. Click here for file

Additional file 2 Complete data of unique and predominant expression of retrogenes and neighboring genes from FlyAtlas. Click here for file

Additional file 3 Distribution of retrogenes (black) and their nearby TEs (red). The two uncertain ROO elements are signed with a question mark. Click here for file
