# Supplementary material for: Evolutionary origin of regulatory regions of retrogenes in Drosophila
Source: BMC Genomics. 2008 May 22;9:241. doi: 10.1186/1471-2164-9-241 (PMC2413143; doi:10.1186/1471-2164-9-241)
Supplement: Additional file 1 — Summary of unique and predominant expression of retrogenes and neighboring genes from FlyAtlas. [file 1471-2164-9-241-S1.pdf]

Additional data file 1. Unique and predominant expression of retrogenes and neighboring genes according to FlyAtlas

| | RETRO GENES Unique | LEFT Unique | RIGHT Unique | RETRO GENES Predom. | LEFT Predom. | RIGHT Predom. |
|---|---|---|---|---|---|---|
| BRAIN | | 1 | 4 | 1 | 2 | 2 |
| HEAD | | 1 | 2 | 1 | 2 | |
| CROP | | | 1 | | | 1 |
| MIDGUT | | 2 | 1 | 2 | 4 | |
| TUBULE | | | 1 | 1 | 1 | |
| HINDGUT | | | 1 | | 1 | 1 |
| OVARY | | | | | 1 | |
| TESTIS | 34 | 18 | 19 | 48 | 29 | 26 |
| MALE ACC. GLANDS | 1 | | | 1 | 2 | |
| Total genes | 94 | 183 | 179 | 94 | 183 | 179 |
| Non-expressed genes | | 5 | 9 | | 5 | 9 |

Non-expressed genes: genes without FlyAtlas entries
