# Supplementary material for: Evolutionary origin of regulatory regions of retrogenes in Drosophila
Source: BMC Genomics. 2008 May 22;9:241. doi: 10.1186/1471-2164-9-241 (PMC2413143; doi:10.1186/1471-2164-9-241)
Supplement: Additional file 3 — Distribution of retrogenes (black) and their nearby TEs (red). The two uncertain ROO elements are signed with a question mark. [file 1471-2164-9-241-S3.pdf]

Additional data file 3. Distribution of retrogenes (black) and their nearby TEs (red).
The two uncertain ROO elements are signed with a question mark.

	mel	*sim*	*sech*	*yak*	*ere*	*ana*	*pseudo*	*per*	*wil*	*vir*	*moj*	*grim*
Upstream												
1) *Fad2*	+	+	+	+	+	+	+	+	-	-	-	-
ROO ?	+	+	+	+	+	+	+	+	+	+	+	+
2) *CG16988*	+	+	+	+	+	+	+	+	+	+	+	+
DNAREP1_DM	+	+	+	-	-	-	-	-	-	-	-	-
3) *CG7094*	+	+	+	+	+	+	+	+	+	+	+	+
DNAREP1_DM	+	+	+	-	-	-	-	-	-	-	-	-
4) *CG2528*	+	+	+	-	-	+	+	+	+	-	-	-
DNAREP1_DM	+	+	+	-	-	-	-	-	-	-	-	-
5) *CG8629*	+	+	+	+	+	+	+	+	+	+	+	+
BS	+	-	-	-	-	-	-	-	-	-	-	-
6) *CG32089*	+	+	+	+	+	+	+	+	+	+	+	+
PROTOP_B	+	-	-	-	-	-	-	-	-	-	-	-
7) *CG11401*	+	+	+	+	+	+	+	+	+	+	+	+
DNAREP1_DM	+	+	+	+	+	-	-	-	-	-	-	-
Downstream												
1) *CG9906*	+	+	+	-	-	-	-	-	-	-	-	-
DMRT1	+	+	+	-	-	-	-	-	-	-	-	-
2) *CG17003*	+	+	+	+	+	+	+	+	+	+	+	+
DNAREP1_DM	+	+	+	-	+	-	-	-	-	-	-	-
3) *CG9564*	+	+	+	+	+	+	+	+	+	+	+	+
ROO	+	-	-	-	-	-	-	-	-	-	-	-
4) *CG8330*	+	+	+	+	+	+	+	+	+	-	-	-
DNAREP1_DM	+	+	+	-	+	-	-	-	-	-	-	-
5) *CG11825*	+	+	+	+	-	-	-	-	-	-	-	-
DNAREP1_DM	+	+	+	-	-	-	-	-	-	-	-	-
6) *CG10104*	+	+	+	+	+	+	+	+	+	+	+	+
DOC	+	-	-	-	-	-	-	-	-	-	-	-
7) *CG12362*	+	+	+	+	+	+	+	+	+	+	+	+
DOC	+	-	-	-	-	-	-	-	-	-	-	-
8) *CG32090*	+	+	+	+	+	+	+	+	+	+	+	+
PROTOP_B	+	+	+	-	-	-	-	-	-	-	-	-
9) *ran-like*	+	+	+	+	+	-	-	-	-	-	-	-
ROO ?	+	+	+	+	+	+	+	+	-	-	-	-
10) *CG11401*	+	+	+	+	+	+	+	+	+	+	+	+
DNAREP1_DM	+	+	+	-	+	+	-	-	-	-	-	-
11) *CG1287*	+	+	+	+	+	+	+	+	+	+	+	+
DNAREP1_DM	+	+	+	-	-	-	-	-	-	-	-	-
12) *CG2512*	+	+	+	+	+	-	-	-	-	-	-	-
DNAREP1_DM	+	+	+	+	+	-	-	-	-	-	-	-
